# Distal muscle weakness is a common and early feature in long-term enzyme-treated classic infantile Pompe patients

**DOI:** 10.1186/s13023-020-01482-w

**Published:** 2020-09-14

**Authors:** J. J. A. van den Dorpel, E. Poelman, L. Harlaar, H. A. van Kooten, L. J. van der Giessen, P. A. van Doorn, A. T. van der Ploeg, J. M. P. van den Hout, N. A. M. E. van der Beek

**Affiliations:** 1 Center for Lysosomal and Metabolic Diseases, Department of Pediatrics, Erasmus MC University Medical Center, P.O. Box 2060, Rotterdam, 3000 CB The Netherlands; 2 Center for Lysosomal and Metabolic Diseases, Department of Neurology, Erasmus MC University Medical Center, P.O. Box 2060, Rotterdam, 3000 CB The Netherlands; 3 Center for Lysosomal and Metabolic Diseases, Department of Pediatric Physiotherapy, Erasmus MC University Medical Center, P.O. Box 2060, Rotterdam, 3000 CB The Netherlands

**Keywords:** Pompe disease, Glycogen storage disease type II, Enzyme replacement therapy (ERT), Muscle function, Distal muscle weakness

## Abstract

**Background:**

Enzyme replacement therapy (ERT; alglucosidase alfa) has improved the prospects for patients with classic infantile Pompe disease considerably. However, over time we noticed that many of these children exhibit distal muscle weakness at an early age, which is in contrast to the primarily proximal and axial muscle weakness in patients with late-onset Pompe disease. This was reason to study the prevalence and severity of distal muscle weakness, and the sequence of muscle involvement over time in patients that had learned to walk under ERT.

**Methods:**

In this prospective, single-center cohort study, we studied 16 classic infantile patients. We used video recordings that were made during regular standardized assessments to investigate distal muscle function (active dorsiflexion of the feet during walking; ability to use a pincer grasp/actively extend the fingers) and proximal muscle function (standing up from a supine position; raising the arms above the head).

**Results:**

Median age at start of ERT was 3.2 months (0.1–5.8 months), median age at study end was 5.6 years (2.9–18.2 years). Six patients (6/16, 38%) initially had no evident signs of distal muscle weakness and developed a gait with active dorsiflexion of the feet. The other 10 patients never exhibited active dorsiflexion of the feet during walking. At study-end two patients showed no loss of distal muscle function. A subset of five patients (5/16, 31%) developed also weakness of the hands, particularly of the extensors of the 3rd and 4th digit.

**Conclusions:**

We found that the majority (14/16, 88%) of patients who had learned to walk exhibited distal muscle weakness of the lower extremities, while a subset (5/16, 31%) also developed weakness of the hands. The distal muscle weakness was often more serious than, and preceded the development of, the proximal muscle weakness.

## Background

Pompe disease (glycogen storage disease type II, OMIM #232300) is a rare metabolic disorder caused by the deficiency of acid α-glucosidase, leading to lysosomal glycogen accumulation in many tissues, particularly in muscle [1]. Pompe disease encompasses a continuous spectrum, varying from a relentlessly progressive classic infantile phenotype to a less progressive, ‘milder’ late-onset phenotype [2, 3]. Classic infantile Pompe disease is characterized by progressive, generalized, muscle weakness manifesting shortly after birth, accompanied by a typical hypertrophic cardiomyopathy. Untreated, patients die before 1 year of age due to cardio-respiratory insufficiency [4, 5]. Limb-girdle muscle weakness and respiratory dysfunction are the hallmarks of the late-onset phenotype, leading to wheelchair and/or respirator dependency in many patients [2, 6–8].

In classic infantile Pompe disease, enzyme-replacement therapy (ERT) with recombinant human acid α-glucosidase (rhGAA, alglucosidase alfa) has shown to improve – ventilator-free – survival, reverse the cardiac hypertrophy, and improve patients’ muscle function, enabling them to reach previously unmet motor milestones such as standing and walking [9–14]. However, remarkably, also many otherwise very ‘good-responding’ ERT-treated classic infantile patients exhibit residual muscle weakness. Over the years, we noticed that this muscle weakness was not limited to the proximal muscles – typically seen in the late-onset phenotype in children and adults – but also involved the distal muscles of the feet [15]. This new phenotype comprising proximal and distal muscle weakness, was also noted by several other authors [16–18].

Presently, it is still unclear which percentage of patients develop this distal weakness, at what age this starts to develop, and how it relates to the development of proximal muscle weakness. Furthermore, it is unknown whether this distal weakness is limited to the lower extremities, or involves also the upper extremities, i.e. the hand musculature. Since we have regularly recorded all motor assessments on video from the start of ERT, we had the unique opportunity to study this intriguing question.

## Results

### Patients

Of the 22 patients with classic infantile Pompe disease that were seen in our Center during the study period, 16 (72%) had learned to walk, and were thus included in this study (Table 1). The most common disease causing variants (www.pompevariantdatabase.nl) were c.del525T (7 patients, 3/7 homozygous) and c.2481 + 102_2646 + 31del (6 patients, 3/6 homozygous). Median age at start of ERT was 3.2 months (0.1–5.8 months), median age at study end was 5.6 years (2.9–18.2 years). Echocardiography showed hypertrophic cardiomyopathy in all patients pre-ERT, with median left ventricular mass index (LVMI) 210 g/m^2^ (range 98–756) and median z-score 19 (range 5–88). During follow-up two patients became ventilator dependent (pat 4 and 11, respective age 2.7 and 2.0 years) after recurrent respiratory infections. Four patients were cross-reactive immunological material (CRIM) negative (pat. 10, 11, 15, 16), one of whom died at the age of 4.4 years due to respiratory failure. The remaining 15 patients were alive at study end.

**Table 1:** Patient characteristics, development of proximal and distal muscle function

		Age				Standing up		Foot dorsiflexion		Raising arms		Pincer grasp	
Pt	Sex	Start ERT (months)	Current (years)	Start dose of ERT	CRIM status	Best achievement (years)*	Last visit	Best achievement (years)*	Last visit	Best achievement (years)*	Last visit	Best achievement (years)*	Last visit
1^a,b,c^	M	4	18	15 /w [r]^g^	+	Gowers (16.4)	Unable to stand up	Mid/forefoot (17.8)	Unable to walk	Normal (16.9)	Not able	Normal (16.9)	No pincer grasp
2^c,d^	M	1	13	20 eow^g^	+	Gowers (10.4)	Unable to stand up	Mid/forefoot (10.6)	Unable to walk	Normal (10.4)	Not able	Normal (5.1)	No pincer grasp
3^c,d^	F	1	12	20 eow^g^	+	Normal (8.1)	Gowers	Heel strike (4.0)	Mid/forefoot	Normal	Normal	Normal	Normal
4^c,d^	M	0	10	20 eow^g^	+	Gowers (2.7)	Unable to stand up	Mid/forefoot (2.7)	Unable to walk	Normal (7.5)	Not able	Normal (6.1)	No pincer grasp
5^c,d^	F	2	8	40 /w	+	Gowers (6.0)	Unable to stand up	Mid/forefoot (6.4)	Unable to walk	Normal	Normal	Normal (7.1)	No pincer grasp
6^d^	F	0	6	40 /w	+	Normal	Normal	Heel strike (4.8)	Mid/forefoot	Normal	Normal	Normal	Normal
7^d^	F	5	6	40 /w	+	Normal	Normal	Heel strike (3.6)	Mid/forefoot	Normal	Normal	Normal	Normal
8^d^	M	4	6	40 /w	+	Normal	Normal	Heel strike (5.0)	Mid/forefoot	Normal	Normal	Normal	Normal
9	M	5	5	40 /w	+	Gowers	Gowers	Mid/forefoot	Mid/forefoot	Normal	Normal	Normal	Normal
10^e^	M	6	5	40 /w^h^	–	Gowers (2.5)	Pull to stand	Mid/forefoot	Mid/forefoot	Normal	Normal	Normal	Normal
11^c^	M	2	4^f^	20 eow	–	Pull to stand (2.1)	Unable to stand up	Mid/forefoot (2.1)	Unable to walk	Normal (3.6)	Not able	Normal (3.1)	No pincer grasp
12^e^	F	4	4	40 /w^h^	+	Normal	Normal	Heel strike	Heel strike	Normal	Normal	Normal	Normal
13^e^	M	3	4	40 /w^h^	+	Normal (2.6)	Gowers	Mid/forefoot	Mid/forefoot	Normal	Normal	Normal	Normal
14	F	3	3	40 /w^h^	+	Normal	Normal	Mid/forefoot	Mid/forefoot	Normal	Normal	Normal	Normal
15	F	2	3	40 /w	–	Normal (1.4)	Gowers	Mid/forefoot	Mid/forefoot	Normal	Normal	Normal	Normal
16	F	5	3	40 /w^h^	–	Normal	Normal	Heel strike	Heel strike	Normal	Normal	Normal	Normal
Median		*3*	*6*										

*Pt* Patient number, *ERT* enzyme-replacement therapy, *CRIM* cross reactive immunological material, *F* female, *M* male, */w* per week, *eow* every other week, [r] recombinant enzyme from transgenic rabbits, switched to alglucosidase alfa at age 5.2 years. ^a-e^ Early motor development described before in: ^a^ van den Hout et al. 2000,^b^ van den Hout et al. 2004 ^c^ van Gelder et al. 2015, ^d^ van Gelder et al. 2016, ^e^ Poelman et al. 2018. ^f^ Age at death. ^g^ Dose was augmented to 40 mg/kg/week at 0.8, 5.5, 9.4 and 2.7 years for patient 1,2,3 and 4 respectively. ^h^ Primary immunomodulation. *Age at which best achievement was lost

### Video analyses of motor function

#### *Lower extremities* (Fig. 1a; Table 1)

Ten patients (63%) showed moderate to severe distal muscle weakness from the beginning. As a result, they never developed a walking gait, with active dorsiflexion of the feet resulting in a heelstrike. The best achievement in these patients was a walking pattern with a mid/forefoot landing. Six patients (37%) initially had no evident signs of distal muscle weakness: they developed a heelstrike-gait at a median age of 3.0 years (range 2.8–3.5 years).

**Fig. 1:**
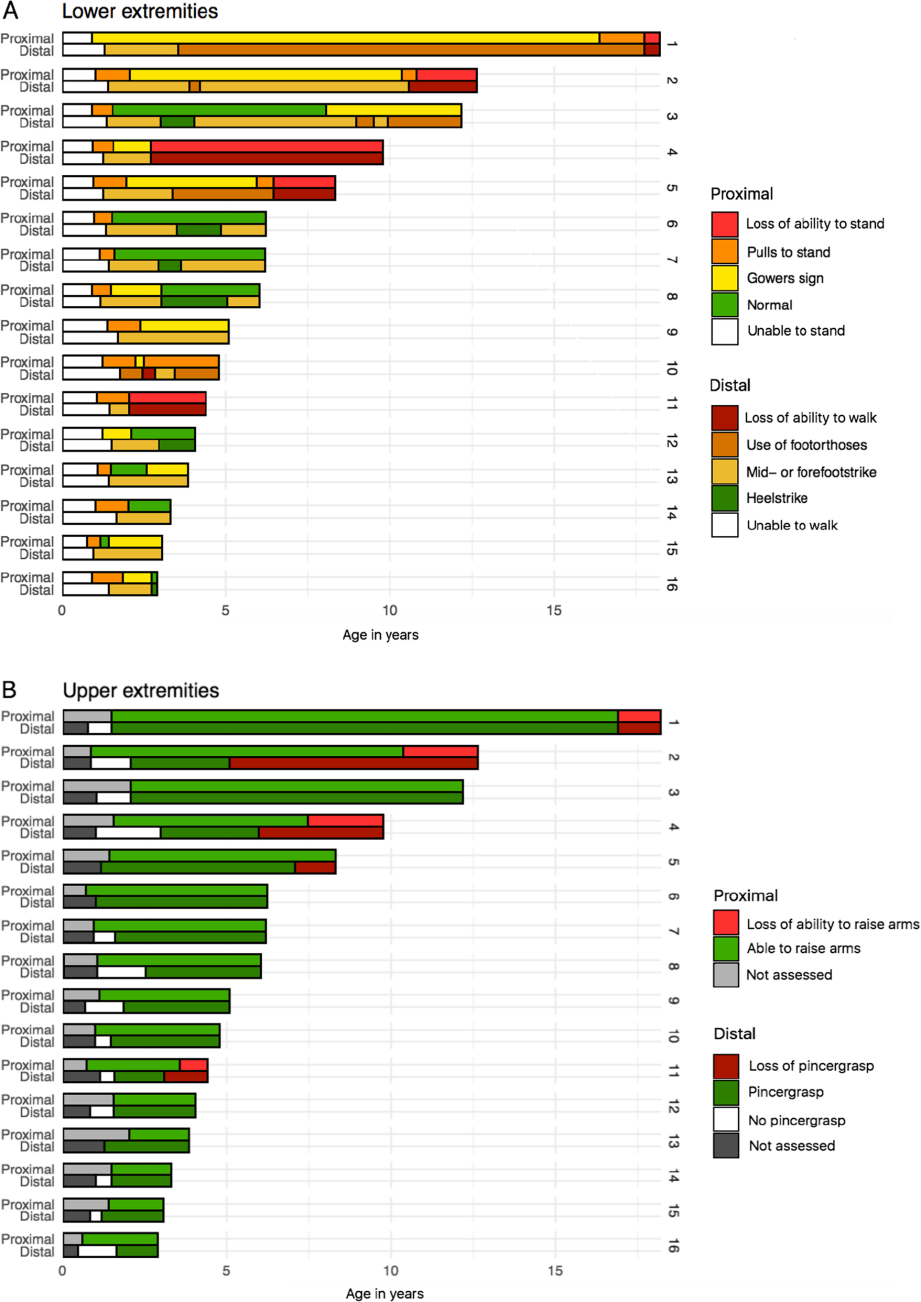
Proximal and distal muscle function in ERT-treated classic infantile Pompe patients. **a** Proximal and distal muscle function of the lower extremities. Upper column: the ability to come to stand from supine, as a parameter for proximal muscle strength of the legs. Lower column: active dorsiflexion of the feet, reflected in the ability to perform a heel-strike during walking. **b** Proximal and distal muscle function of the upper extremities. Upper column: raising arms above head as parameter for proximal muscle function. Lower column: the ability to use a pincer grasp, while performing fine motor tasks

With regard to proximal muscle function, nine patients (56%) were at any point in time able to rise from a supine position without any visible difficulty or use of external support. The other seven patients (44%) always showed some signs of proximal muscle weakness, reflected by a Gowers sign or Trendelenburg gait. There were no differences regarding the age at which patients had learned to stand and walk between the patients with or without proximal muscle weakness.

The six patients who initially had no distal muscle weakness also had normal proximal muscle function in the beginning. At the end of the study, four of these patients had reverted to a mid/forefoot landing during walking: three had lost their heelstrike-gait (at age 3.6, 4.0 and 5.0 years) but still had no signs of proximal muscle weakness, while one had developed distal muscle weakness (at age 4.0 years), before also developing proximal muscle weakness (at age 8.1 years). Only two of the 16 patients (13%) had no distal weakness at their last assessment at the ages of 2.9 and 4.0 years. They also had no signs of proximal muscle weakness (Table 1).

Of the other 10 patients, at study end, one had normal proximal muscle function but impaired ankle dorsiflexion, four were still able to walk but with evidence of both proximal and distal muscle weakness, while five had lost the ability to stand and walk and had become wheelchair bound due to severe overall muscle weakness.

#### Dosing and CRIM status

Only one of the five patients (20%) who were initially treated with a lower dose of recombinant enzyme achieved a walking pattern without apparent distal and proximal muscle weakness, while five of the 11 patients (45%) treated with 40 mg/kg/week initially showed no muscle weakness of either distal or proximal muscles.

One of the CRIM negative patients (1/4, 25%; treated with 40 mg/kg/week from the start, additional immunomodulation) and 5/12 (42%) of the CRIM positive patients initially achieved a walking pattern with a normal heelstrike-gait.

#### Upper extremities (Fig. 1b; Table 1)

**Fig. 2:**
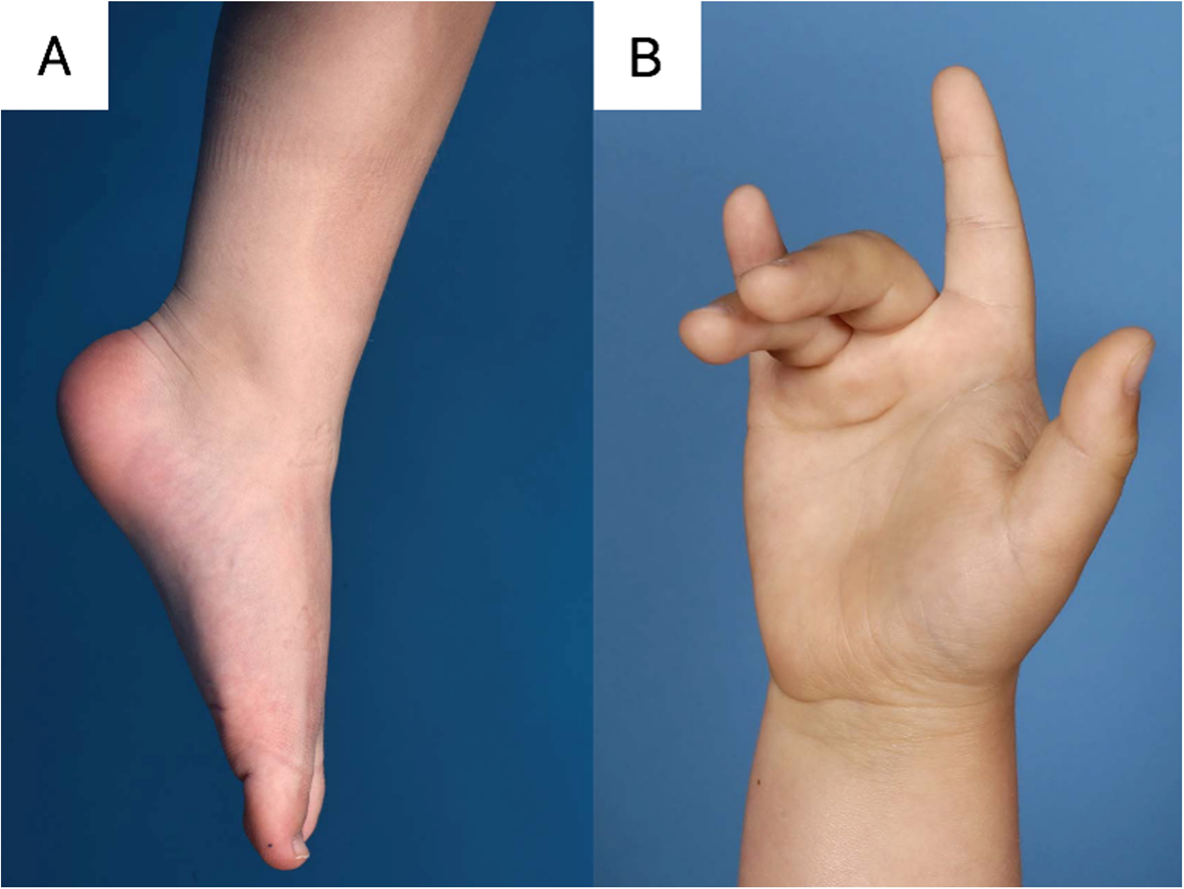
Distal muscle weakness of the feet and hands in ERT-treated classic infantile Pompe patients. **a** Severe weakness of foot dorsiflexor muscles, patient was asked to perform active dorsiflexion of the foot. **b** Weakness of finger extensor muscles, with characteristic positioning of the 3rd and 4th digit. Picture was taken at maximal finger extension

All patients acquired the ability to fully raise their arms above the head and obtained normal fine motor skills, as was evidenced by a normal pincer grasp when picking up small objects (achieved at median age 1.6 years, range 1.0–3.0). During follow-up, three patients developed profound finger extensor weakness mainly of the third and fourth digit (patients 2, 4, and 5; Fig. 2b). These three patients also developed weakness of the upper arms at a later stage. Two other patients also developed some distal muscle weakness reflected by a more crude grip using the whole hand, one of whom had no signs of upper-arm weakness, while in the other patient loss of proximal and distal arm function had coincided with a fracture of the clavicle. The other 11 patients had exhibited no visible impairment of upper-arm and hand function at the end of follow-up. Weakness of the upper extremities was seen only in patients with severe proximal and distal weakness of the lower extremities at an earlier stage.

### Other motor features

#### Early motor function development

Patients were able to stand and walk independently at the respective median ages of 1.0 (range 0.8–1.4) and 1.4 years (range 1.0–1.8), which was within the 90th percentile of healthy age-matched controls in 8/16, 50% (standing) and 13/16, 81% (walking) of patients.

Functional motor development, as reflected by their AIMS and BSIDII scores, showed a gradual improvement after start of ERT. At their last BSIDII evaluation, 9/16, 56% of patients reached an age-equivalent score, reflecting normal development (Data not shown).

#### Supportive measures

Due to decreased ankle dorsiflexor strength (Fig. 2a), nine patients eventually needed supportive measures. Seven patients (pat 2–5, 7, 13, 15) used splints at night to increase ankle mobility and prevent contractures (start use splints between 2.5 and 8.0 years), while five patients (pat 1–3, 5, 10) used ankle foot orthoses to provide support when walking (start use foot orthoses between 1.8 and 9.0 years). Three patients (pat 2, 3, 5) used both splints and ankle foot orthoses. With these measures, we were able to maintain at least 90 degree ankle dorsiflexion angle in ambulant patients. None of the patients underwent surgery for contractures or other foot deformities. One patient (pat 2) used a hand splint (from age 5.6 years) to support hand function and prevent contractures.

## Discussion

Long-term survival of classic infantile Pompe patients treated with ERT has unveiled a specific phenotype. We studied the occurrence and sequence of involvement of distal and proximal muscle weakness in 16 patients with classic infantile Pompe disease. Although all patients learned to walk, we found that: 1) 88% of patients developed distal muscle weakness of the lower extremities at any point in time, 2) 31% of patients developed also weakness of the hands, particularly of the extensors of the 3rd and 4th digit, and 3) patients exhibited a specific pattern of muscle involvement, in which distal muscle weakness of the lower extremities was more serious than and preceded proximal muscle weakness; likewise, weakness of the hands occurred earlier than that of the upper-arms.

Early motor development showed significant improvement after start of ERT, which enabled patients to achieve motor milestones such as standing and walking. Many patients even learned to walk at a normal age compared to their peers.

However, over time, 14 of the 16 patients (88%) developed distal weakness of the legs. Importantly, most patients never developed a walking pattern with active foot dorsiflexion (10/16; 63%): six patients (37%) initially developed a normal heelstrike-gait, four of whom displayed secondary progressive weakness of the distal muscle groups. Only 2 patients – still relatively young (2.9 and 4.3 years) – had a normal heelstrike-gait at the end of follow-up. In our study, all patients above the age of 5 years exhibited distal muscle weakness. From a functional point of view, the distal muscle weakness was in most cases even more severe than the accompanying proximal muscle weakness. Compared to healthy peers, the development of a mature gait with a heelstrike was delayed in all patients (≈1.5 years against 3.0 years) [19].

There are only few reports in the literature on the occurrence of distal muscle weakness in long-term treated classic infantile patients. In one retrospective study [17] nine out of 11 treated classic infantile patients, median age 8.0 years (range 5.4–12.0), developed dorsiflexor weakness of the feet. The age at which this distal weakness occurred, or the pattern in which distal and proximal weakness developed, was not reported. Two patients did not show any dorsiflexor weakness (age 5.4 and 8.2 years at study-end). A recent case study reports subacute – over several months – development of dorsiflexor weakness at an older age (6.5 years), attributed to progressive myopathy and possibly also minor peripheral nerve involvement [20].

To our knowledge, weakness of the hand musculature has not been reported in earlier studies. We found that weakness of the upper extremities, including weakness of hand musculature was less prominent and occurred only in patients with severe weakness of lower extremities. Three of the five patients with hand-weakness had a particular positional tendency with flexion of the third and fourth finger. This may result from the fact that third and fourth digit are controlled by only one finger extensor muscle, contrary to the other digits [21].

We found that motor outcome differed between patients. Several factors, including age at start of treatment [9, 10], ERT dose [15] and CRIM status [22, 23], have shown to be of influence on survival and motor outcome. In our study, patients who were treated initially with a lower dose more frequently showed persistent weakness of foot dorsiflexion, compared to patients treated with 40 mg/kg/week. This better outcome in the higher-dose group is in accordance with our earlier findings [15]. The influence of CRIM status on motor outcome was less apparent. However, it should be noted that the follow-up of the CRIM negative patients was shorter.

The severe and early distal weakness that we have found is discrepant with the predominant limb-girdle and axial weakness seen in children and adults with late-onset Pompe disease. Involvement of dorsiflexor muscles of the feet or specific involvement of the hand musculature does typically not occur in those patients until end-stage disease [6, 7, 24, 25]. Only one case of adult Pompe disease has been described, in which weakness of wrist extensor muscles was one of the presenting symptoms [26].

The pathophysiological process underlying the distal muscle weakness is still not fully understood. Several factors might play a role, including extensive involvement of distal muscles in the myopathic process [20, 27], and potential neurogenic involvement due to glycogen storage in the central [28–30] and peripheral nervous system [20, 31, 32]. Detailed radiological, electrodiagnostic and histological studies in larger numbers of patients are needed to provide a better understanding of the underlying etiology.

Our study had several limitations. Despite a relatively large cohort size of classic infantile patients, the absolute number of subjects is still small. Although we would have liked to provide a more quantitative measure of muscle strength, such as manual muscle testing or hand held dynamometry, to strengthen our opinion on the degree of distal versus proximal weakness, these measures are notably unreliable in young children. Additionally, a formal gait analysis studying kinematics of additional muscle joints could have provided more detailed information.

Since we recorded all assessments on video from the beginning, we were able to precisely look at when the distal muscle weakness developed. Loss of ankle dorsiflexion results in an unstable (steppage) gait, which may increase the risk of falling and, consequently, occurrence of fractures. Furthermore, distal muscle weakness may lead to loss of joint mobility resulting in development of contractures. Therefore, awareness and early recognition of distal weakness is important for timely application of supportive measures such as splints and foot orthoses.

## Conclusion

Although many patients now learn to walk under ERT-treatment, the majority develops distal muscle weakness, most prominent of the feet but also of the hand musculature, which is often more serious than, and occurs mostly prior to development of proximal muscle weakness.

## Methods

### Patients and study design

This study is part of a prospective, open-label, cohort study on the long-term effects of enzyme-replacement therapy in classic-infantile Pompe disease, conducted at the center for Lysosomal and Metabolic Diseases of Erasmus MC University Medical Center, the designated center of expertise for Pompe disease in the Netherlands. Patients were included if 1) they had a confirmed diagnosis of classic-infantile Pompe disease, defined as manifesting muscle weakness within 6 months of birth, a hypertrophic cardiomyopathy, and two deleterious disease-causing variants in the acid-α-glucosidase gene (*GAA)*, and 2) had learned to walk independently. The earliest data that were used for the purpose of this study are from 1999, database lock was December 31st 2016. Informed consent was obtained from the parents of all patients. From 2009 onward, we treated newly diagnosed patients with recombinant human enzyme (rhGAA; alglucosidase alfa) at a dose of 40 mg/kg/week from the start. Before that, some patients had initially been treated by recombinant enzyme from transgenic rabbits or with a lower dose of 20 mg/kg/2 weeks; for all patients initially receiving 20 mg/kg/2 weeks, between 2009 and 2014 their dose of alglucosidase alfa was increased to 40 mg/kg/week. Immunomodulation in an ERT-naïve setting with Rituximab (RTX), Methotrexate (MTX) and intravenous immunoglobulins (IVIG) was initiated in 2012, in newly diagnosed patients older than 2 months of age at time of diagnosis. (See for details Poelman et al., 2018) [33].

### Procedures

#### Video analyses of motor function

To investigate the occurrence and severity of distal muscle weakness, and the sequence of muscle involvement, we used video recordings of the regularly performed standardized clinical assessments (AIMS, BSID-II, Quick motor function test (QMFT), Six-minute walk test (6MWT) and timed tests: running 10 m, standing up from supine position (TT)) to construct an overview of the motor development of the patients through time. This resulted in a 4-h summary (= total for all patients) of video material obtained from 122 h of film, comprising 481 clinical assessments. Video recordings were analyzed every 3 months until the age of 1 year, twice a year between 1 to 3 years of age, and once a year from the age of 3 onwards.

From these video recordings we assessed the following to investigate distal muscle function: (1) active dorsiflexion of the feet during walking resulting in a heel-strike (yes / no) and; (2) weakness of the hands and fingers: ability to use a pincer grasp (yes / no) and being able to actively extend the fingers (yes / no). Proximal muscle weakness was evaluated by assessing (1) coming to stand from a supine position (without difficulty / visible difficulty but without external support (Gowers maneuver) / only with external support (e.g. chair/table) / not able) and (2) raising arms above the head in a straight position (able / not able).

All video material was scored by three independent examiners with experience in pediatrics and/or neuromuscular disorders. If no consensus was reached, results were discussed, and agreed upon in a consensus meeting (JvdD and NvdB).

#### Other motor features

Additional investigations to describe overall early motor function development comprised 1) motor milestones (independent sitting, standing, and walking), 2) the Alberta Infant Motor Scale (AIMS), and 3) Bayley Scales of Infant Development II (BSID-II). Established reference values were used to compare patients with age-matched controls [34–36]. Furthermore, we collected information on the timing and type of supportive measures for foot dorsiflexion and prevention of joint contractures, as well as the use of hand splints to support hand functioning.

### Statistical analyses

Due to the small number of patients no comparative statistics were applied. Descriptive statistics, including median and range, were used to summarize demographic and clinical data (SPSS for Windows (version 24, IBM Corp, Armonk, NY). For comparison with healthy peers, established reference values were used.

## Data Availability

The dataset is summarized in the figures and table. The raw data used and/or analyzed during the study are available from the corresponding author on reasonable request. For reasons of privacy, the video material is not publicly available.

## Abbreviations

6MWT: Six minute walking test
AIMS: Alberta Infant Motor scale
BSID-II: Bayley Scales of Infant Development II
CRIM: Cross-reactive immunologic material
ERT: Enzyme replacement therapy
GAA: Acid-α-glucosidase
QMFT: Quick motor function test
rhGAA: Recombinant human alpha-glucosidase
TT: Timed tests
